# An Alternative Way to Reach the Epicardial Focus of the Left Ventricular Tachycardia in a Patient with Non-ischemic Cardiomyopathy

**DOI:** 10.1016/s0972-6292(16)30736-7

**Published:** 2014-03-12

**Authors:** Dursun Aras, Serkan Topaloglu, Serkan Cay, Ozcan Ozeke, Goksel Cagirci, Ugur Canpolat

**Affiliations:** Yuksek Ihtisas Heart-Education and Research Hospital, Ankara, Turkey

**Keywords:** coronary sinus, endocardial, epicardial, ventricular tachycardia

## Abstract

We report a case of a 69-year-old male with non-ischemic cardiomyopathy, having drug- and antitachycardia pacing-refractory ventricular tachycardia resulted in multiple ICD shocks. The sustained and intractable ventricular arrhythmia was mapped and ablated with the aid of the three-dimensional electroanatomic mapping system, initially performed but unsuccessful from the endocardial site then performed successfully from the epicardial site via the coronary sinus.

## Introduction

In some instances, catheter ablation from the epicardial aspect of the ventricles is needed although the majority of ventricular tachycardia (VT) is located and ablated in the right or left ventricular endocardium [1]. Epicardial VT ablation has been shown to improve outcomes in patients with non-ischemic cardiomyopathy (NICM) and is required for more than 30% of VTs in these patients [2,3].

## Case presentation

**Figure 1:**
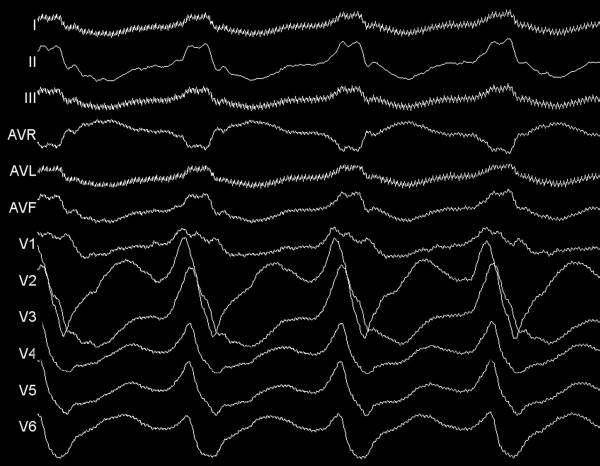
Twelve-lead ECG showing the VT at a paper speed of 100 mm/sec.

**Figure 2:**
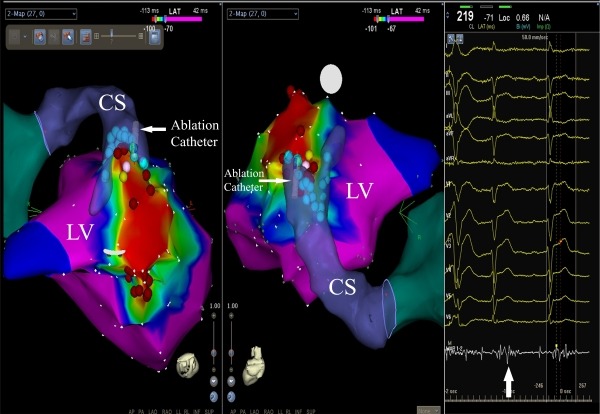
Carto 3 images showing both endocardial (red dots) and epicardial (light blue dots) ablation sites in the basal posterolateral wall of the left ventricle in the straight cranial (left panel) and posteroanterior (middle panel) views. Yellow point shows the pacing site resulted in fully matched 12-derivation ECG pattern with VT (see Figure 3). White point shows the epicardial zone where fairly fragmented diastolic potentials in the MAP recording line (white arrow at the far bottom of the right panel) were obtained. CS, coronary sinus; MAP, mapping; LV, left ventricle.

**Figure 3:**
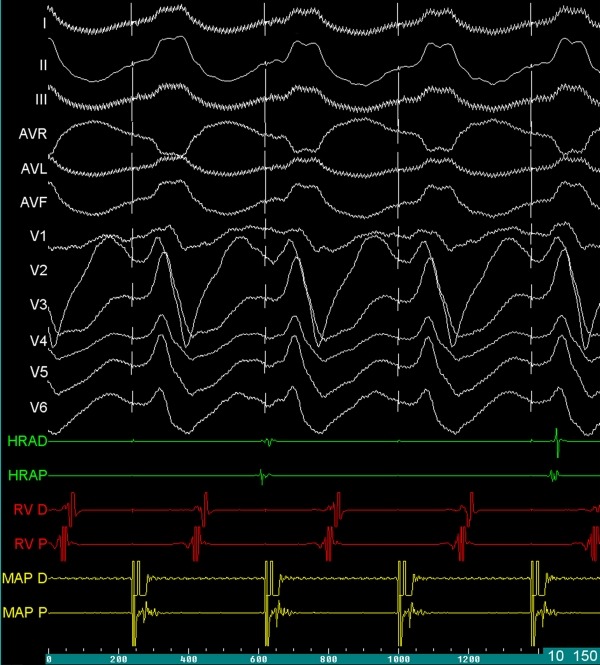
Twelve-lead ECG showing fully matched 12-derivation ECG pattern with VT at a paper speed of 100 mm/sec. The pacing site was from the coronary sinus (yellow point in Figure 2). HRA, high right atrium; MAP, ablation catheter; RV, right ventricular apex.

**Figure 4:**
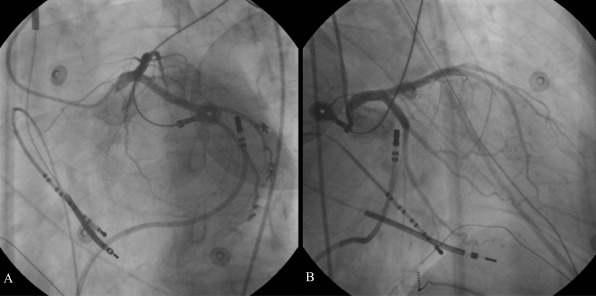
Epicardial ablation of VT focus originating from the basal posterolateral region of the left ventricle through the CS in the left (450) (A) and right (300) (B) oblique projections during VT. Dual coil ICD electrode and decapolar electrode catheter were seen in the right ventricular apex. CS, coronary sinus.

A 69-year-old male patient with NICM was referred to our department for multiple antiarrhythmic drug- and antitachycardia pacing-refractory ventricular tachycardia (VT) episodes resulted in electrical storm and depletion of the battery of his VVI-ICD. No secondary abnormalities causing the tachycardia were detected. Surface electrocardiogram during VT revealed a possible focus of the basal posterolateral wall of the left ventricle (Figure 1). Voltage-mapping during sinus rhythm demonstrated no identifiable scar tissue. Activation-mapping during the tachycardia using the Carto 3 electroanatomic mapping system (The Carto 3 System, Biosense Webster, Belgium) pointed out the same anatomic region for VT focus (Figure 2). In addition, tachycardia zone was marked by pacing and entrainment maneuvers that the earliest endocardial recordings were obtained as - 45 ms compared to QRS onset on activation mapping, pace mapping at this site generated a 12-lead ECG morphological match, and during entrainment maneuvers post-pacing interval was equal to tachycardia cycle length as 400 ms, and stimulus-QRS interval was equal to diastolic potential-QRS interval as 190 ms demonstrating the location was near the central portion of the isthmus (stimulus QRS/tachycardia cycle length = 48%). Endocardial radiofrequency energy applications to this region prolonged the VT cycle length without eliminating the tachycardia (red dots in Figure 2). Before entering the pericardial space, close anatomic relation with this endocardial region was through the coronary sinus (CS) epicardially. Irrigated tip ablation catheter (Navistar® Thermocool®, Biosense Webster, Belgium) was introduced and placed laterally in the CS. Endocardially obtained earliest local activation times were also detected on the epicardial site during the VT. Pace mapping from this zone resulted in matching of all 12-derivation ECG pattern of the VT as in the endocardial site (Figure 3). In addition, clear fragmented diastolic potentials were detected in sinus rhythm (white arrow in Figure 2). Before introducing radiofrequency energy, selective left system coronary angiography was performed because of close proximity to the circumflex artery (Figure 4). The safety distance from the artery was enough. Afterwards, multiple radiofrequency energy applications (450C, 25 W, 15 ml/min) on and around the pointed out zones finally stopped the VT. Programmed stimulation was repeated at least 30 min after the last delivery of radiofrequency energy to confirm the noninducibility of the VT. Easily inducible VT has never been induced anymore. Six-month follow-up period was asymptomatic.

## Discussion

Although automaticity and triggered activity may responsible from the electrophysiological mechanisms of VT in NICM, the proven histopathological relation between cardiomyopathic changes including myocyte hypertrophy and fibrosis and, the inducibility of VT with extrastimuli may also suggest the susceptibility to reentrant arrhythmias. Most of the cases with VT can be successfully mapped and ablated from the endocardial site. When this is not possible, however, invasive percutaneous epicardial route may be required to map and ablate VT. Previously published several recent reports have demonstrated that the venous system of the heart seems to be alternative route for mapping and ablation of VTs originating from an epicardial region [4-6]. Li et al. [7] have demonstrated that ECG patterns of ventricular arrhythmias arising from the different parts of the venous system including great cardiac vein and anterior interventricular vein were different, pace mapping and ablation from these sites were effective and safe. The venous system of the ventricles should be kept in mind when ablation attempts from the endocardial site are unsuccessful. Some distinct features obtained from surface ECG during arrhythmia can differentiate the epicardial origin of VT from the endocardial VTs. These are the pseudodelta (the interval from the earliest ventricular activation to the earliest fast deflection in any chest lead), the intrinsicoid deflection time (the interval from the earliest ventricular activation to the peak of the R wave), and the maximum deflection index (the intrinsicoid deflection time/QRS duration). A duration of pseudodelta of ≥34 ms and duration of intrinsicoid deflection of >85 ms have sensitivities of 83% and 87%, and specificities of 95% and 90%, respectively [8]. A maximum deflection index of ≥0.55 has also high sensitivity and specificity, 100% and 99%, respectively [9]. Similar to these findings, Li et al [7] have found that the prolonged intrinsicoid deflection time >70 ms and maximum deflection index >0.54 suggested the epicardial site with high sensitivity (83% for both) and specificity (96% and 97%, respectively). In our case, we measured the pseudodelta as 50 ms, the intrinsicoid deflection time as 96 ms, and maximum deflection index as 0.59 suggesting an epicardial site for the VT. Before attempting to enter the pericardial space, the venous system of the ventricles such as coronary sinus and its branches should be mapped to identify the origin of VT in patients having pseudodelta, prolonged intrinsicoid deflection time and higher maximum deflection index. In addition, fragmented diastolic potentials, earliest activation times, pace mapping for ≥11/12 ECG match, and entrainment maneuvers should be used because invasive percutaneous epicardial way can cause rare but important life-threatening complications. This route also requires experience.
